# Exploitation of various physio-morphological and biochemical traits for the identification of drought tolerant genotypes in cotton

**DOI:** 10.1186/s12870-023-04441-2

**Published:** 2023-10-23

**Authors:** Tahreem Arif, Muhammad Tanees Chaudhary, Sajid Majeed, Iqrar Ahmad Rana, Zulfiqar Ali, Hosam O. Elansary, Ihab Mohamed Moussa, Sangmi Sun, Muhammad Tehseen Azhar

**Affiliations:** 1 Department of Plant Breeding and Genetics, University of Agriculture, Faisalabad, 38040 Pakistan; 2 Federal Seed Certification and Registration Department, Ministry of National Food Security and Research, Islamabad, 44090 Pakistan; 3 Center of Advance Studies/Center of Agricultural Biochemistry and Biotechnology, University of Agriculture, Faisalabad-38040, Pakistan; 4 Department of Plant Production, College of Food & Agriculture Sciences, King Saud University, P.O. Box 2460, Riyadh, 11451 Saudi Arabia; 5 Department of Botany and Microbiology, College of Science, King Saud University, P.O. Box 2455, Riyadh, 11451 Saudi Arabia; 6 Department of Biotechnology, Chonnam National University, Yeosu, 59626 Korea; 7 School of Agriculture Sciences, Zhengzhou University, Zhengzhou, 450000 China

**Keywords:** Biochemical assays, Breeding, Cotton, Drought, Germplasm, Relative leaf water content

## Abstract

**Background:**

Drought is one of the limiting factors for quality and quantity of cotton lint in tropical and sub-tropical regions. Therefore, development of drought tolerant cotton genotypes have become indispensable. The identification of drought tolerant genotypes is pre-requisite to develop high yielding cultivars suitable for drought affected areas.

**Methods:**

Forty upland cotton accessions were selected on the basis of their adaptability and yield. The collected germplasm accessions were evaluated at seedling stage on the basis of morphological, physiological and biochemical parameters. The experiment was conducted under controlled conditions in greenhouse where these genotypes were sown under different levels of drought stress by following factorial under completely randomized design. The data were collected at seedling stages for root and shoot lengths, relative leaf water content, excised leaf water losses, peroxidase content and hydrogen peroxide concentrations in leaf tissues.

**Results:**

The biometrical analysis revealed that germplasm is significantly varied for recorded parameters, likewise interaction of genotypes and water stress was also significantly varied. The cotton germplasm was categorized in eight clusters based on response to water stress. The genotype Cyto-124 exhibited lowest H_2_O_2_ content under drought conditions, minimum excised leaf water loss under stress environment was exhibited by genotypes Ali Akber-802 and CEMB-33. Overall, on the basis of morphological and biochemical traits, SL-516 and Cyto-305 were found to be drought tolerant. Genotypes 1852 − 511, Stoneville 15–17 and Delta Pine-55 showed low values for root length, peroxidase activity and higher value for H_2_O_2_ contents. On the basis of these finding, these genotypes were declared as drought susceptible.

**Conclusion:**

The categorization of cotton germplasm indicating the differential response of various parameters under the control and drought stress conditions. The recorded parameters particularly relative leaf water contents and biochemical assays could be utilized to screen large number of germplasm of cotton for water deficit conditions. Besides, the drought tolerant genotypes identified in this research can be utilized in cotton breeding programs for the development of improved cultivars.

**Supplementary Information:**

The online version contains supplementary material available at 10.1186/s12870-023-04441-2.

## Introduction

Cotton is the most important natural fiber crop. Its fibers are mainly used in textile industry. It belongs to genus *Gossypium* which comprises 54 species [1]. *Gossypium hirsutum* is most widely cultivated species which accounts for >90% cotton growing area of the world. Cotton production in Pakistan during 2020-21 showed significant decline of approximately 22.8% over the previous year. This decline was due to numerous factors. One of the important factors was climate change phenomenon includes,  extensive dry periods and hot environmental conditions [2]. Thus, the requirement of abiotic stress tolerant cotton cultivars has increased [3]. It is a crucial time and sustainable approach to improve the genetics of cultivated cotton to enhance the stress tolerance abilities because it is estimated that drought affected terrestrial land will be doubled by the end of 2 century [4].

The fresh biomass of plants is comprised of 85–90% water, which plays an important role in various physiological processes including plant growth, development and metabolism. The degree of damage to the cotton crop varies at different levels of drought stress. Overall, drought stress exerts adverse effects on plant biomass production, i.e., decrease in leaf area, shoot and root weight, stem thickness and lint percentage. Drought stress also leads to physiological changes like closure of stomata, reduction in relative leaf water content, decrease in stomatal conductance, cease of capillary movement, assimilation stops and reduction in leaf water potential. It reduces the yield and fiber quality due to disruption in cellular homeostasis.

The concentration of reactive oxygen species (ROS) increases under increased level of drought stress conditions. When ROS production is higher than plant’s ability to scavenge excess ROS and maintain an optimal concentration than rapid increase of ROS occur in the cells (a state known as oxidative stress). Increased level of ROS is not desirable for normal growth of plants and may alter its physiological and metabolic processes. It also causes high lipid peroxidation and decline in Rubisco and photo-chemicals efficiency which leads to poor crop growth [5]. ROS are produced in various organelles i.e. mitochondria, chloroplast and peroxisomes of plant cells. In addition, ROS are also the byproducts of metabolic processes. ROS, including hydrogen peroxide (H_2_O_2_), superoxide anion (O^2−^), hydroxyl radical (OH^−^), and singlet oxygen (O_2_), each has a characteristic of half-life and oxidizing potential [6, 7]. Antioxidant defense enzymes like peroxidase (POD) play role in maintaining the balance among ROS and related scavenging elements. Plants have various antioxidative mechanism i.e. enzymatic and non-enzymatic components that regulate the synthesis of ROS synthesis, scavenging of cellular damage. The types of non-enzymatic components includes ascorbic acids, flavanoids, glutathione, α-tocopherol, carotenoids, lipids, and phenolic compounds, have proven to pay their role efficiently by reducing the activity of ROS through the involvement of H_2_O_2_ [8]. ROS works like sword which exerts oxidative stress according to the levels (high, medium to low), which mediates the signal transduction that assists in maintaining the cellular homeostasis and also helpful in acclimatization under to stressful conditions. [9]. Studies have shown that anti-oxidative activity is associated with increased stress tolerance in plants [6].

Previous experiments revealed the differential responses among cultivars for morphological and physiological attributes under various levels of drought stress [10]. Although drought stress is common in arid areas, but irrigated lands may also suffer from dry weather due to high temperature and limited water availability in rivers and canals. Drought stress issues can be resolved by adapting various stress coping/mitigation strategies like modifying irrigation methods, cultural practices and developing tolerant crop varieties [11]. Various studies have been conducted on physiological characteristics related to drought (relative leaf water content, excised leaf water loss, leaf water temperature, water use efficiency, leaf water potential, stomatal size and frequency, osmotic potential, stomatal conductance, *etc*.) and have been suggested as screening criteria for selecting drought resistant plants [12]. Although drought tolerant germplasm exists in cotton but there was a dire need to screen it under various levels of drought stress and related morphological, physiological and molecular traits. Therefore, this research work was planned to identify drought tolerant genotypes from the germplasm. The identified genotypes can be further utilized in breeding programs. In this research, we also evaluated the response of some obsolete cultivars under drought stress conditions. The outcomes of this study might be a contribution in various breeding programs being executed for the development of drought tolerant genotypes of cotton.

## Materials and methods

The experiment was conducted in greenhouse of the Department of Plant Breeding and Genetics, University of Agriculture Faisalabad (Pakistan) during 2020–2021. The germplasm of upland cotton was selected based on their diverse genetic background, adaptability to local conditions, yield and wide cultivation across the country (Table 1).

**Table 1:** List of genotypes of upland cotton used in the experiment for characterization against various levels of water stress conditions.

Sr. No	Genotype	Sr. No	Genotype	Sr. No	Genotype
1	Cyto-178	15	Chandni-95	29	1841 − 449
2	Cyto-161	16	1856 − 536	30	1859 − 558
3	Cyto-179	17	1852 − 511	31	Tarzon-1
4	Cyto-177	18	1855 − 533	32	Stamp-81
5	Cyto-124	19	1854 − 528	33	Stone Ville-108
6	Cyto-517	20	1845 − 472	34	Stone Vile 15–17
7	Cyto-313	21	1847 − 483	35	Ali Akber-802
8	Cyto-305	22	1863 − 577	36	Ali Akber-703
9	SL-516	23	1853 − 570	37	Delta Pine-55
10	Cyto-608	24	1866 − 598	38	4 F
11	Cyto-164	25	1842 − 455	39	CRIS-134
12	FC-4245	26	1843 − 461	40	CRIS-508
13	VH-305	27	1839 − 438		
14	CEMB-33	28	1840 − 441		

### Assessment of plant material for drought stress tolerance

The experiment was comprised of three treatments, i.e., 50%, 75% and 100% of field capacity to find drought tolerant genotypes. Genotypes were sown in polythene cups (30 × 14 cm size) in two replications following factorial under complete randomized design (RCBD). Seeds were soaked in tab water overnight before sowing. In each replication, three cotton seeds were sown at approximately 2 cm depth in a polythene cup filled with sand. Two out of three seedlings were thinned after germination to keep one healthy seedling. Temperature in glasshouse was maintained at ~ 35 °C during day time and ~ 27 °C during night time. Electric bulbs were used to maintain day light intensity at 2,400 lx. Nitrogen in the form of 0.2 g urea was supplied in each cup after 14 days of sowing and seedlings were initially irrigated for better growth and development. The weight of soil moisture at field capacity was calculated as difference between soil weight after drainage and soil weight after oven drying for 105 °C for 24 h. Later, pot water holding capacity of all the treatments were measured based on dry soil and wet soil weight with the help of moisture meter (TDR-100). Polythene bags were weighed in grams on daily basis and seedlings were watered accordingly. The experiment was continued until fourth main stem leaf was appeared, and then plants were uprooted. [13]. Then data were recorded for following traits at 45 days old seedlings for root length (cm), shoot length (cm), relative leaf water content, excised leaf water loss, peroxidase (U mg^− 1^ protein) and hydrogen peroxide (µmol g − 1 (FW) [11].

.

### Root length and shoot length

Seedlings were uprooted gently from sand to avoid any breakage of roots. The roots were separated by cutting the intersection of root and shoot. Roots were washed with tab water to remove sand. The length of root was recorded in cm using measuring tape. Finally mean values of root length of each genotype in each treatment were calculated for statistical analysis. Shoot length was also recorded in cm with measuring tape and mean values of shoot length were also calculated for biometrical analysis.

### Relative leaf water content

Three-leaf samples were taken from selected plants under control and drought conditions. Fresh weight of leaves was taken using electronic balance. Leaves were dipped in water overnight for acquiring turgid pressure. After turgidity was attained, the leaves were weighed and kept at room temperature (25 °C) for one hour for drying. Samples were kept in an oven at 70 °C for 72 h for dry weight. Relative leaf water content was calculated using formula outlines by Barrs and Weatherly [14].

1 $$RWC=\left[\frac{Fresh weight-dry weight}{Turgid weight-dry weight}\right]\text{x} 100$$

### Excised leaf water loss

Fresh leaf weight was recorded using electronic balance ALE-223. Leaves were kept at room temperature for 24 hours for wilting. Then weight of wilted leaves was noted. For dry weight calculation, the samples were kept again in an oven at 70 °C temperature for 72 h. Excised leaf water loss was calculated according to method proposed by Clarke and McCaig [15]

2 $$ELWL=(Fresh weight-wilted weight)/Dry weight$$

### Peroxidase content

The leaf tissues were ground in pestle and mortar using 0.05 M sodium phosphate buffer then centrifuged at 10,000 rpm for 20 min and supernatant was taken in micro centrifuge tube. The reaction mixture of 3 ml was prepared by mixing the equal volume of guaiacol and H_2_O_2_. Then reaction mixture was poured into enzyme extract. Finally, absorbance was measured at 470 nm using Nano Drop Spectrophotometer 2000 according to protocol as suggested by Fielding and Hall [16].

### Hydrogen peroxide

H_2_O_2_ content was estimated by following the method of Bernt and Bergmeyer [14]. Fresh leaf tissues were immediately kept at -80 °C in freezer after harvesting. Then 0.5 g tissue was homogenized with 5 ml of 0.1% (W/V) Trichloroacetic Acid (TCA) in sterilized pestle and mortar. To scale down the amount, weighed 0.1 g (100 mg) of fresh tissue was homogenize with 1ml of 0.1% (W/V) TCA in a microfuge^®^20 by crushing the tissue at low temperature by placing it on ice bath. The remaining mixture was centrifuged at 12,000 × g for 15 min, then 0.5 ml of the potassium phosphate buffer (pH 7.0) and 1 ml of 1 M potassium iodide (KI) was added to 0.5 ml of the supernatant. Finally, the mixture was vortexed and absorbance was measured at 390 nm.

### Statistical analysis

Genetic variability among genotypes were assessed using analysis of variance with factorial design using method as purposed by Steel et al. [17]. Biplot and cluster analysis were performed with the help of *SPSS v.19* and *STATISTICA v.5.0*, *respectively*. The analysis is interpreted in subsequent sections of this manuscript.

## Results

### Screening of germplasm

#### Analysis of variance

Analysis of variance revealed that significant differences among genotypes for drought tolerance abilities. The interaction of genotypes with different levels of drought stresses (G×T) was significant (Table 2). Mean square values for recorded traits were also significant under control and drought stress conditions (P ≤ 0.01).

**Table 2:** Mean square values of forty upland cotton accessions for various traits under various levels of droughts stress

SOV	DF	RL	SL	RLWC	ELWL	POD	H_2_O_2_
**Genotypes**	39	5.590**	10.53**	78.2**	0.379**	10.032**	0.012**
**Treatments**	2	363.139**	1660.02**	18282.7**	3.390**	0.686**	0.033**
**Genotypes × Treatments**	78	0.639**	3.03**	39.6**	0.082**	8.005**	0.012**
**Error**	120	0.139	0.25	8.0	0.019	3.001	0.000
**Total**	239						

Where, SOV: Sources of variations; DF: degree of freedom; RL: root length; SL: shoot length; RLWC: relative leaf water content; ELWL: excised leaf water loss; POD: peroxidase; H_2_O_2_: hydrogen peroxide

### Mean comparison of various parameters under normal and stress conditions

The average mean values from three drought stress levels revealed that SL-516 genotype had maximum root length (10.2 cm) followed by genotypes 1840 − 441 (7.13 cm), 1842 − 455 (8.35 cm) and 1840 − 441 (8.43 cm). These genotypes performed better under the provided conditions. Genotype CEMB-33 showed minimum root length followed by 1852 − 511 and Stone-ville-15-17 with average values of 3.52 cm, 5.06 and 7.36 cm, respectively (Table S1). Maximum shoot length was observed in SL-516 and Cyto-305 with average values of 14.49 cm and 14.13 cm, respectively (Table S 1.1). The genotype SL-516 and Cyto-305 appeared as drought tolerant, while 1852 − 511 as drought susceptible because it showed poor response for recorded parameters under various levels of drought stress. Under control condition, maximum relative leaf water content was exhibited by genotype 1843 − 461 (82.5%) followed by Cyto-517 (82%) while minimum relative leaf water content was found in Cyto-178 (66.33%) and 1839 − 483 (63.33%). Under drought conditions, high values for relative leaf water content were found in 1863 − 577 (63.5%), 1855 − 533 (61.1%), SL-516 (56.83%) and Cyto-517 (54.83%), while accessions with lower values for relative leaf water contents were 1847 − 483 and Cyto-178 with mean value of 33.7 and 33.16, respectively (Table S 1.2). Likewise, variation in relative leaf water content was also observed in drought tolerant and susceptible cultivars. The genotypes 1843 − 461, Cyto-517 and SL-616 showed high relative leaf water contents while 1839 − 438, Delta Pine-55 and Cyto-178 showed low values for relative leaf water content under drought conditions. Maximum excised leaf water loss was shown by the genotypes namely, Cyto-517 (2.00) and 1863 − 577 (1.98) whereas, minimum excised leaf water loss for this trait was exhibited by Ali Akber-802 (0.47) and CEMB-33 (0.54) under drought stress conditions. Genotypes with minimum values of excised leaf water loss were Cyto-177, Ali Akber-802 and 1854 − 528 with average values of 0.6, 0.26 and 0.5, respectively (Table S 1.3).

Mean values for peroxidase enzyme activity (POD) at various drought stress levels are provided in table S 1.4. Results revealed that POD for all accessions varied from each other and ranged from (10 to 14.7 U mg^-1^ protein). At 75% moisture, POD was relatively increased and ranged from (10 to 17.5 U mg^-1^ protein). Lowest value for POD was recorded for the genotype Cyto-161 (10 U mg^-1^ protein) and highest value was observed in genotype 1839 − 483 (17.5 U mg^-1^ protein). At 50% moisture, maximum POD was recorded for genotype 1866 − 598 (22 U mg^-1^ protein) and minimum for genotype VH-305 (10 U mg^-1^ protein) (Table S 1.4). Mean values of H_2_O_2_ at 100%, 75% and 50% moisture levels are given in Table S 1.5. Results revealed that H_2_O_2_ contents for all the accessions under control conditions differed from each other and ranged between 0.12 and 0.36 µmol g^− 1^ FW) (Table S 1.5). At 75% moisture; lowest H_2_O_2_ content were recorded for Cyto-124 (0.12 µmol g^− 1^ FW) and highest values for this parameter were observed in genotype 1854 − 528 (0.38 µmol g^− 1^ FW). At 50% moisture, maximum H_2_O_2_ content were recorded for Cyto-517 (0.36 µmol g^− 1^ FW) and minimum for Cyto-177 (0.12 µmol g^− 1^ FW).

### Principal component analysis

**Fig. 1:**
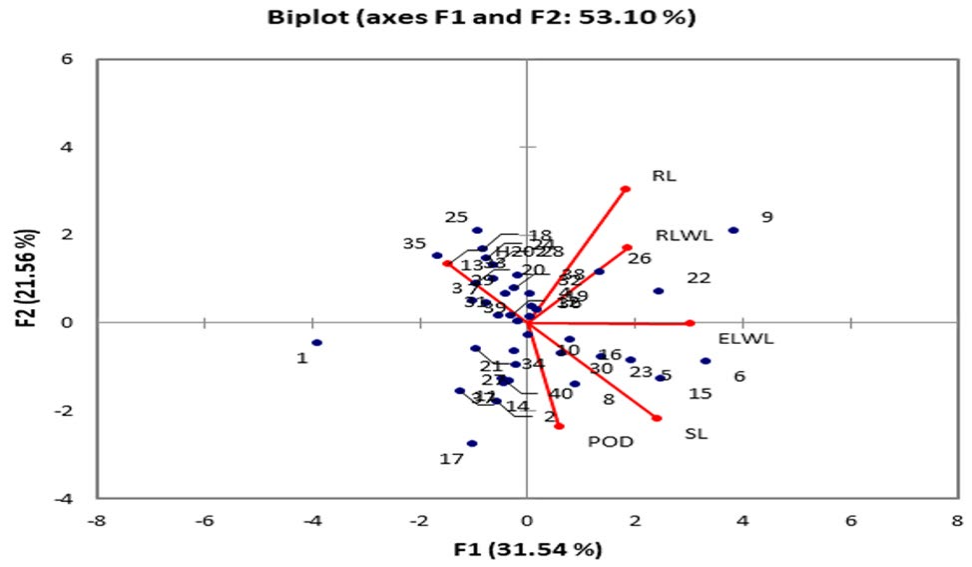
Biplot analysis for various traits of 40 cotton genotypes under 100% moisture level

Three principal components (PCs) showed values more than one under normal conditions. The first three PCs contributed 0.712% of the total variability among genotypes assessed for seedling traits. The PC-I contributed maximum towards the variability (0.305%) followed by PC-2 (0.214%) (Table S2). The trait H_2_O_2_ showed negative factor loadings (-0.328%) on PC-I and PC-6 (-0.049%) while all other traits had positive loadings. In PC-2 three traits RL (0.573%), RLWC (0.393%) and H_2_O_2_ (0.330%) exhibited maximum positive factors loading while other parameters SL (-0.449%), ELWL (-0.029%) and POD (-0.454%) had negative loadings on PC-2. A PC biplot given in Fig. 1 indicates that variables and genotypes are drawn on plot as vectors. The distance of variables with respect to PC1, PC2 and PC3 showed contribution of these variables in variation among accessions. The biplot analysis revealed that the RL and RLWL were lied close to each other on biplot and are also positively correlated, whereas another positive correlation between two traits SL and ELWL were reported in this study (Fig. 1).

**Fig. 2:**
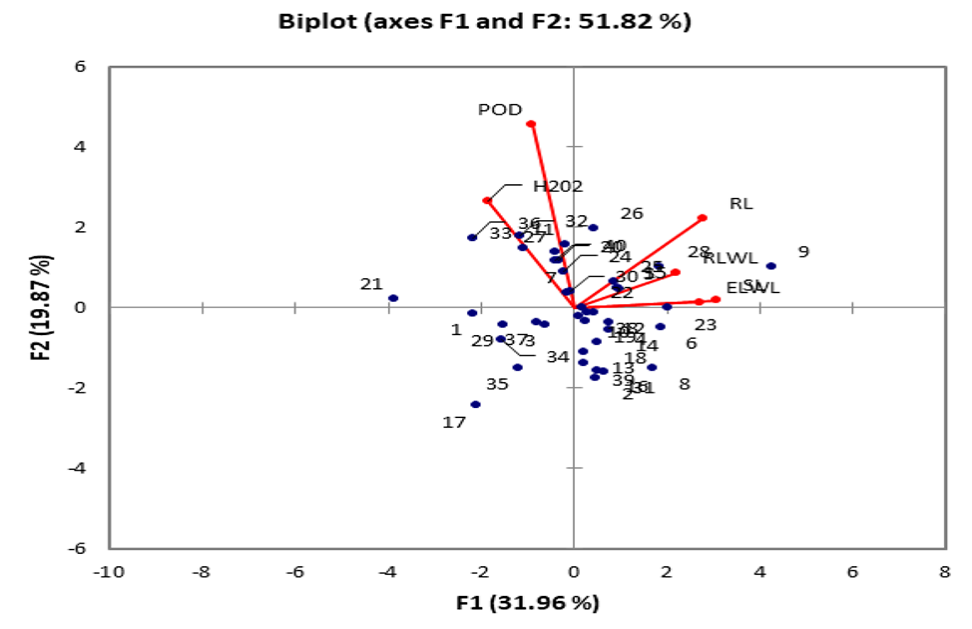
Biplot analysis for various traits of 40 cotton genotypes under 75% moisture level

At 75% moisture level, two out of six principal components showed more than 1.0 eigen values. These two PCs contributed 0.5% of the total variability. The PC-1 contributed maximum towards the variability (0.313%) followed by PC-2 (0.187%) as shown in table S3. The traits such as POD (-0.094%) and H_2_O_2_ (-0.310%) showed negative factors loading on PC-1 while rest of the traits had positive loading. Root length (-0.307%), ELWL (-0.031%), POD (-0.849%) and H_2_O_2_ (-0.381%) exhibited negative loadings while shoot length (0.078%) and RLWC (0.182%) exhibited positive loadings on PC-2. Root length, RLWL, ELV and shoot length lied close to each other towards the direction of PC-1, which reveals positive association among them. On the basis of these traits the genotype No. 9 (SL-516) appeared to be drought tolerant (Fig. 2).

**Fig. 3:**
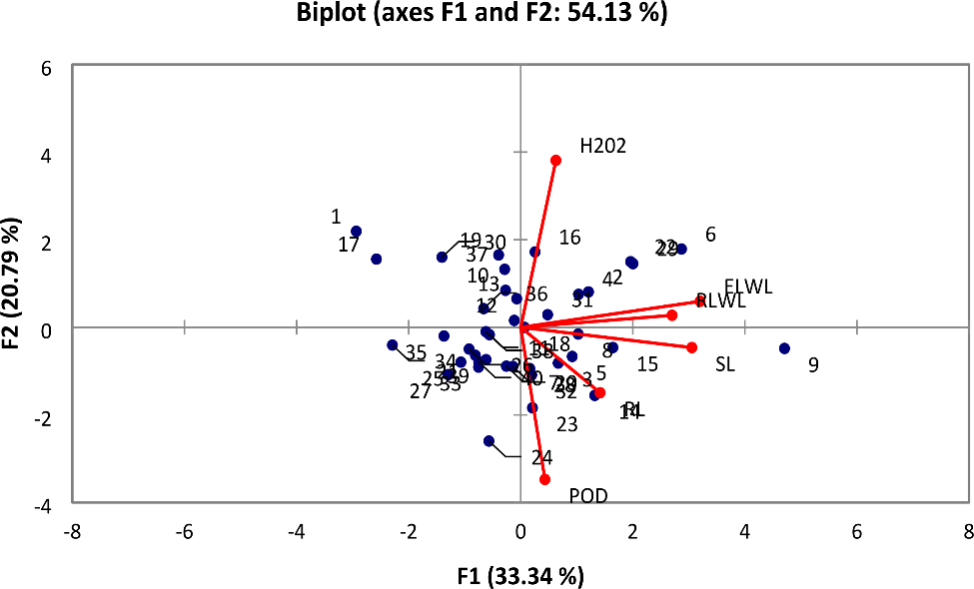
Biplot analysis for various traits of 40 cotton genotypes under 50% moisture level

At 50% moisture level, three out of six PCs had eigen values more than one. These three PCs contributed 0.7% of the total variability. The contribution of PC-1 was maximum towards the variability (0.327%) followed by PC-2 (0.203%) and PC-3 (0.170%) (Table S4). All traits showed positive factors loading in PC-1. The traits such as RLWC (-0.071%), ELWL (-0.126%) and H_2_O_2_ (-0.707%) showed negative factor loadings on PC-2 while all other traits had positive loadings while RLWC (-0.343%), ELWL (-0.226%) and POD (-0.392%) showed negative factor loadings on PC-3 (Fig. 3).

### Cluster analysis

#### Cluster analysis at 100% moisture level

**Fig. 4:**
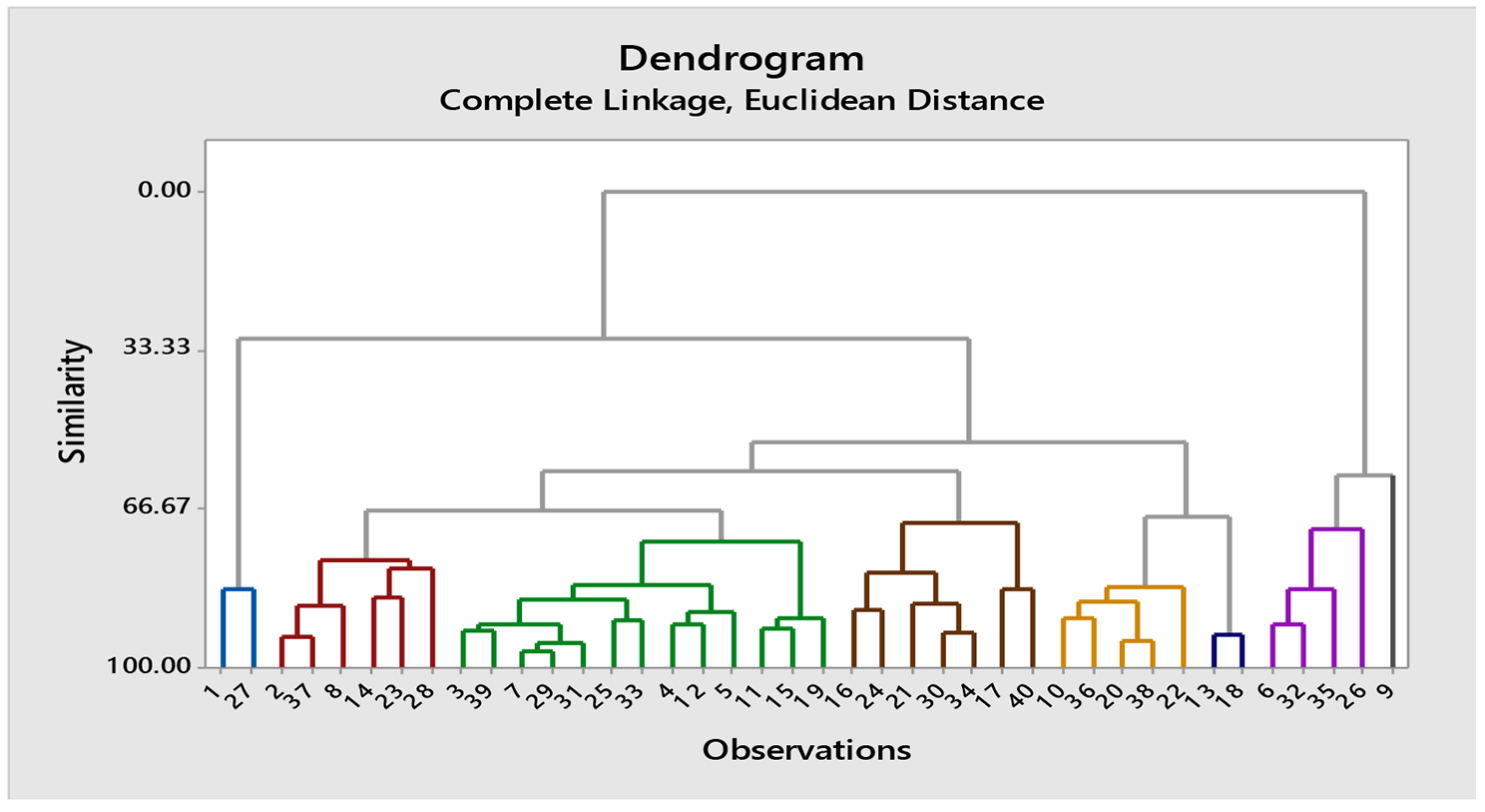
Dendrogram of cotton genotypes resulting from cluster analysis under normal conditions

Genotypes were grouped into 8 clusters on the bases on mean values of parameters included in this study. At 100% moisture level, the maximum mean value of root length (8.8 cm) was observed in genotypes of cluster 1 while cluster 5 showed minimum mean value for root length (4.45 cm). Similarly,  genotypes in cluster 1 have maximum mean for shoot length (18.6 cm), while cluster 8 exhibited minimum mean value for this parameter (14.4 cm). The RLWC has higher mean value in cluster 2 than cluster 8 genotypes. Genotypes of cluster 1 has highest mean value for ELWL (1.91) while genotypes in cluster 8 have minimum mean value (0.81) for this trait. Maximum mean value for POD (16.25 U mg^-1^ protein) was observed in cluster 2 genotypes. Similarly, maximum mean for H_2_O_2_ contents (0.25 µmol g^− 1^ FW) were observed in cluster 8 and minimum mean value (0.15 µmol g^− 1^ FW) in cluster 1 genotypes (Table S5). Dendrogram of cotton genotypes resulting from cluster analysis under 50% moisture level is given below (Fig. 4).

#### Cluster analysis at 75% moisture level

**Fig. 5:**
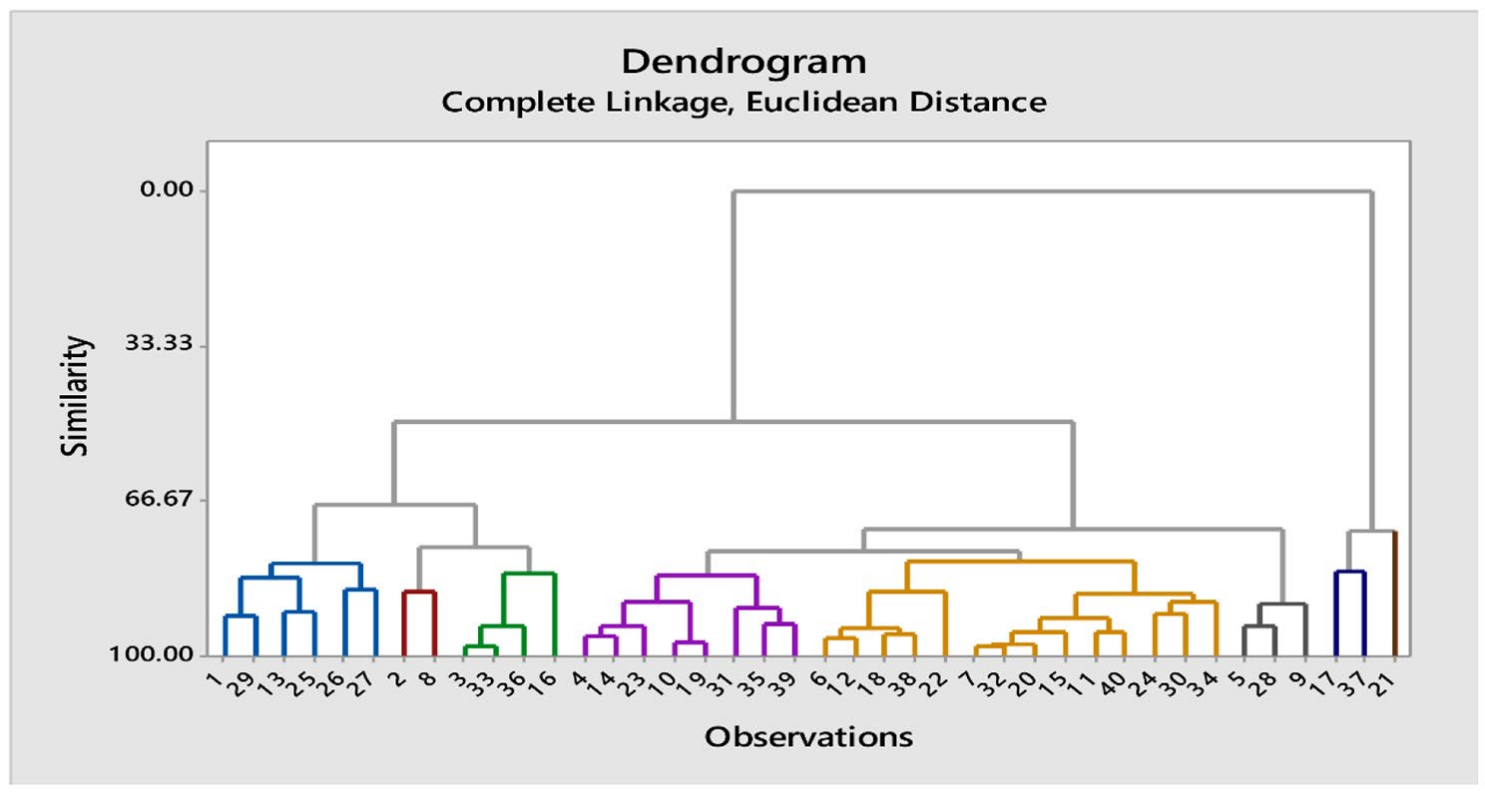
Dendrogram of cotton genotypes resulting from cluster analysis under 75% moisture level

At 75% moisture level, the software divided the genotypes in eight clusters on the basis of trait values. (Fig. 5; Table 3). Highest mean value of root length (8.03 cm) was observed for genotypes in cluster 3 while genotypes of cluster 1 showed minimum mean root length (5.2 cm). Clusters 3 and 7 showed high mean value for shoot length (13.8 cm) and cluster 1 exhibited minimum mean value (8.2 cm). The RLWC has high mean value (60.2%) in cluster 4, while cluster 1 revealed lowest mean value for this parameter. Highest mean value for ELWL was exhibited by genotypes of cluster 7. Maximum mean value for POD (15 U mg^-1^ protein) exhibited by genotypes of cluster 1 and minimum mean value (10.5 U mg^-1^ protein) in cluster 7 (Table S6).

**Table 3:** Cluster membership of various cotton genotypes at various moisture level

Clusters	Name of genotypes in each cluster at 100% moisture level	Name of genotypes in each cluster at 75% moisture level	Name of genotypes in each cluster at 50% moisture level
Cluster 1	SL-516	1847 − 483	SL-516
Cluster 2	1843 − 461, Ali Akbar 802, Stamp-81, Cyto-517	Delta pine-55, 1852 − 511	Cyto-517, Tarzon-1, 1856 − 536, VH-305, Cyto-608, Cyto-177
Cluster 3	1855 − 533, VH-305	SL-516, 1840 − 441, Cyto-124	Stamp-81, 1841 − 449, Chandni-95, Cyto-313, Cyto-124, Cyto-179
Cluster 4	1863 − 577, 4 F, 1845 − 472, Ali Akbar 703, Cyto-608	Stone ville 15–17, 1859 − 558, 1866 − 598, Cris-508, Cyto-164, Chandni-95, 1845 − 472, Stamp-81, Cyto-313, 1863 − 577, 4 F, 1855 − 533, FC-4245, Cyto-517	1866 − 598, Cemb-33
Cluster 5	Cris-508, 1852 − 511, Stone-ville 15–17, 1859 − 558, 1847 − 483, 1866 − 598, 1856 − 536	Cris-134, Ali Akber-802, Tarzon-1, 1854 − 528, Cyto-608, 1853 − 570, Cemb-33, Cyto-177	1859 − 558, Ali Akber703, 1863 − 577
Cluster 6	1854 − 528, Chandni-95, Cyto-164, Cyto-124, FC-4245, Cyto-177, Stone-ville 108, 1842 − 455, Tarzon-1, 1841 − 449, Cyto-313, Cris-134, Cyto-179	1856 − 536, Ali Akber-703, Stone ville-108, Cyto-179	Stone ville 15–17, 1847 − 483, 1852 − 511, Ali Akber-802, 1854 − 528, FC-4245, 1839 − 438, Stone ville-108, Cyto-164
Cluster 7	1840 − 441, 1853 − 570, Cemb-33, Cyto-305, Delta pine-55, Cyto-161	Cyto-305, Cyto-161	4 F, 1840 − 441, 1842 − 455, Cris-134, Cris-508, 18845-472, 1853 − 570, 1855 − 533, Delta pine-55, Cyto-305, Cyto-161
Cluster 8	1839 − 438, Cyto-178	1839 − 438, 1843 − 461, 1842 − 455, VH-305, 1841 − 449, Cyto-178	1843 − 461, Cyto-178

#### Cluster analysis at 50% moisture level

**Fig. 6:**
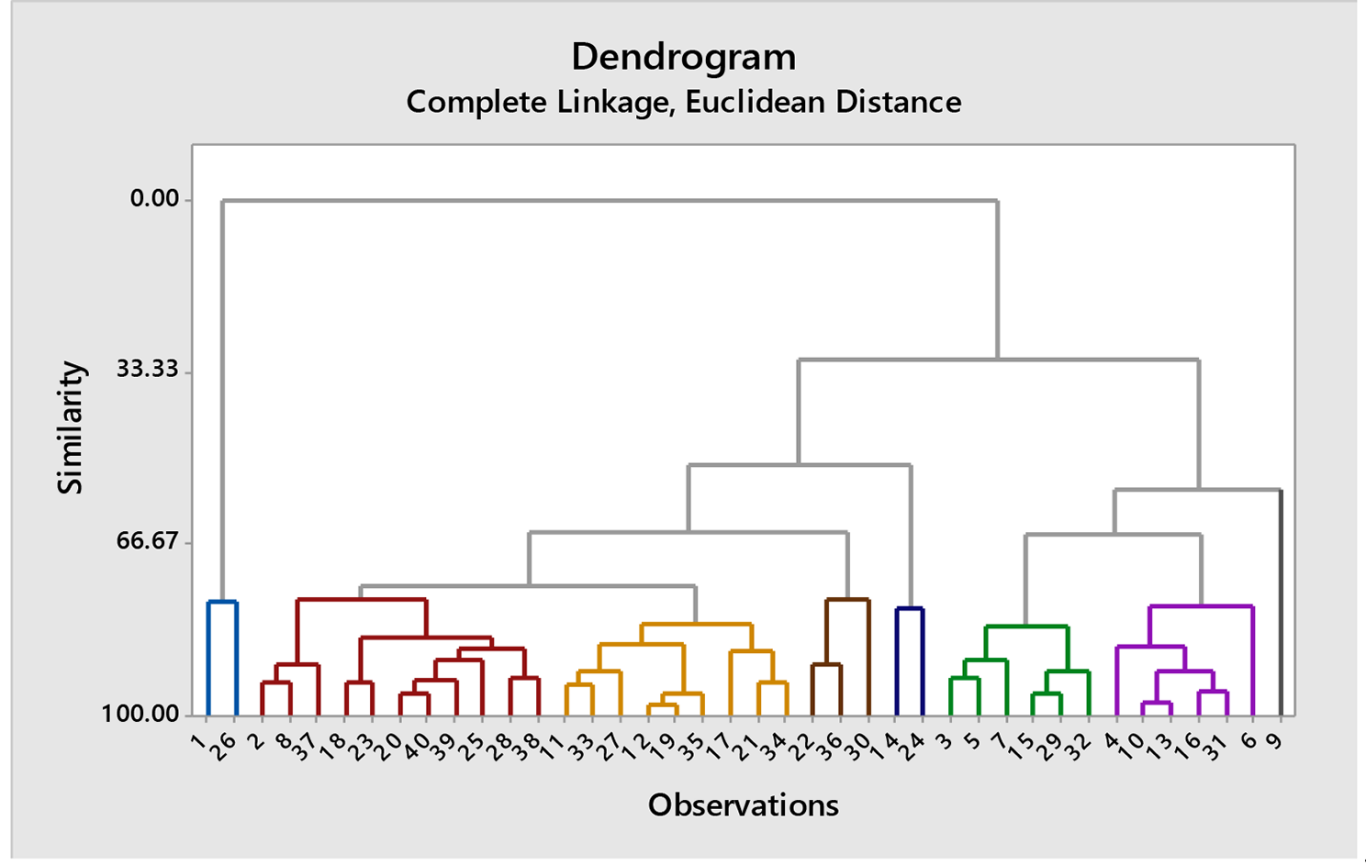
Dendrogram of cotton genotypes resulting from cluster analysis under 50% moisture level

At 50% moisture level, 40 cotton accessions were grouped into 8 clusters on the basis of their mean values of the studied traits (Table 3). The maximum mean value of root length (12.79 cm) was observed for genotypes of cluster 1 while genotypes of cluster 4 showed lowest mean value (7.9 cm) for this attribute. Likewise, cluster 1 genotypes also exhibited maximum mean value for shoot length (14.4 cm). The mean value for RLWC were highest (56.8%) for cluster 1 and lowest (34.8%) for cluster 8 genotypes. Genotypes in cluster 1 showed high mean value for POD (15.5 U mg^-1^ protein) and lowest mean value for this antioxidant was observed for genotypes fall cluster 5 (10.5 U mg^-1^ protein). Highest mean value for H_2_O_2_ contents were observed in cluster 8 genotypes (0.32 µmol g^− 1^ (FW) while lowest in cluster 3 accessions (Table S7). Dendrogram of cotton genotypes resulting from cluster analysis under 50% moisture level is given below (Fig. 6).

## Discussion

Climatic changes have resulted in drought and heat stresses which are adversely affecting the production and quality of seed cotton [18]. Less availability of water and distorted patterns of rainfalls are starting points of droughts [19]. Rising of global temperature is also causing shortage of water due to more use and rapid transpiration/evaporation. Seed viability, germination and developmental stages of seedlings are highly effected due to drought stress. Various studies showed the presence of negative impact of drought on germination and seedling growth [20]. The affects of drought are more adverse on shoot tissues of cotton as compared to root. Various plant parameters namely, length of seedling, numbers of nodes, leaf area, and dry weights of stem and leaves were significantly reduced in drought as compared to controll conditions. Similarly, length of root was also effected in water deficit conditions in contrary to control plants. [21]. The affects of water stress on roots and shoots were more than leaves of same accessions. The effects water were las on medium size roots as compared to small roots due to their vigor. It was concluded that medium roots are more important for growth in water stress conditions [22]. The analysis of data revealed that water stress significantly decreases the root length. Plants were unable to maintain their internal turgor pressure under water shortage, which results in slow rate of cell division and cell elongation. It is also reported that cell divides rapidly due to mitosis in root tips but this process slows down due to loss of turgor pressure in root tissues [23]. At seedling stage, length of root gives a fair and logical idea about overall growth of root at lateral stages [24]. Therefore, accessions, CEMB-33 with less root length (3.52 cm) followed by 1852 − 511 and Stone Ville 15–17 (5.06 and 7.36 cm) respectively, at seedling stage are categorized as susceptible to drought stress.

Shoot length is reported as an important parameter to assess the effect of drought in crop plants [25–27]. The shoot length significantly reduce under the increased level of drought stress [28]. Under drought conditions, the nutrients translocated in root cells. These cells help the plants to uptake water and nutrients from lower surface of soil, while over accumulation of nutrients in these cells due to less growth of shoot tissues resulted in poor plant development [29]. The presence of more RLWC is a good indicator to gauge the status of water in leaves and assess the drought tolerance ability in plants [30]. It has been reported that accessions with higher RLWC are more productive under drought stress [31]. RLWC in leaves have also been reported as a direct indicator of water content under water deficit conditions [32]. ELWL is also a drought stress related trait because under normal condition plant do not make these adjustments. Hence, ELWL showed negative association with other traits under normal conditions. It is because ELWL is an energy consuming process and plant have to make these adjustments at the cost of energy required for its proper growth and development [33]. Various genotypes exhibited differential response of ELWL under drought levels due to variation in thickness of cuticle layer [5].

Drought stress enhances the production of ROS in the cells. Plant leaves are badly effected by the increased concentrations of ROS such as H_2_O_2_ which leads to oxidative damage to the cells due to lipid peroxidation [9]. High levels of oxidative stress also cause denaturation of important cellular protein especially the proteins found in cell membranes which results into alteration of its permeability [34]. Therefore, leakage of electrolyte starts from cell membrane which results into decrease in cell membrane thermostability. In this way, drought stress combines with heat stress cause more damage to plant productivity. Activity of peroxidase enzyme was higher in those genotypes which performed better for other attributes than those with lower values of root and shoot lengths. Similar to other antioxidants, peroxidase scavenged hydrogen peroxide to maintain its optimal level in the cell. The concentration of ROS increases in cotton genotypes due to drought stress, likewise genotypes having higher levels of POD were considered as tolerant and optimal level of hydrogen peroxide was found in those genotypes which further confirms the scavenging activity of peroxidase. Similar findings were also reported in cotton, chickpea [35] and wheat [36].

Principal component analysis (PCA) analysis categorized the extent of variation in characters in the experimental material. The genetic resources are utilized by dividing the total variance into its components [37]. In this experiment, three out of six principal components had eigen value > 1 in control and drought conditions, i.e., 75% & 50%. The principal components I had maximum contribution towards the variability followed by component II. Zafar et al. [10] and Zahid et al. [5] also reported significant contribution of first principal components in total variability of cotton germplasm. Cluster diagrams was generated based on three water levels which divided the cotton genotypes into various clusters. Where, cluster 1 was comprised of genotypes having maximum values for all the parameters associated with drought stress tolerance while cluster 8 revealed minimum values for the recorded traits. Ayana and Bekele [38] and Rabbani et al. [39] also reported that relationship between various clusters were made based on origin of genotypes and agronomic parameters studied in *Brasica juncea* and *Pisum sativum*, respectively. Similar variations in clusters generated under different levels of drought stress were reported by Amna et al. [40] and Maruti et al. [41]

## Conclusion

Drought is one of the major constraints of low yield in crops and posing threat to the future of agriculture. It is necessary to develop drought tolerant as well as high yielding varieties of upland cotton on priority basis. The potential of identified drought tolerant genotypes (SL-516 and Cyto-305) could be further evaluated for yield and fibre quality related traits by sowing on drought affected areas. These genotypes can be utilized in breeding programs for the development of improved germplasm. Such germplasm would be helpful for breeders and could enhance the area of cultivation of cotton that will ultimately increase in cotton production.

### Electronic supplementary material

Below is the link to the electronic supplementary material.

Supplementary Material 1

## Data Availability

The datasets used and/or analyzed during the current study available from the corresponding author on reasonable request.
