# Supplementary material for: Exploitation of various physio-morphological and biochemical traits for the identification of drought tolerant genotypes in cotton
Source: BMC Plant Biol. 2023 Oct 23;23:508. doi: 10.1186/s12870-023-04441-2 (PMC10591375; doi:10.1186/s12870-023-04441-2)
Supplement: Supplementary file 1 — Supplementary Material 1 [file 12870_2023_4441_MOESM1_ESM.docx]

**Table S1. Mean values of root length of cotton genotypes grown under three moisture levels.**

| **Sr.**  **No.** | **Genotypes**  **Name** | **Root Length** | | | | **Sr.**  **No.** | **Genotypes**  **Name** | **Root Length** | | | |
| --- | --- | --- | --- | --- | --- | --- | --- | --- | --- | --- | --- |
|  |  | **100%** | **75%** | **50%** | **Mean.**  **value** |  |  | **100%** | **75%** | **50%** | **Mean.**  **value** |
| 1 | Cyto-178 | 4.4 | 6.09 | 7.4 | 5.9 | 21 | 1847-483 | 4.73 | 5.2 | 8.2 | 6.0 |
| 2 | Cyto-161 | 4.1 | 5.3 | 7.8 | 5.7 | 22 | 1863-577 | 7.5 | 6.08 | 9.7 | 7.7 |
| 3 | Cyto-179 | 5.04 | 5.65 | 8.6 | 6.4 | 23 | 1853-570 | 7.05 | 7.7 | 10.4 | 8.4 |
| 4 | Cyto-177 | 3.99 | 6.5 | 9.26 | 6.5 | 24 | 1866-598 | 5.3 | 7.3 | 10.2 | 7.6 |
| 5 | Cyto-124 | 5.25 | 7.26 | 10.12 | 7.5 | 25 | 1842-455 | 5.5 | 8.35 | 11.1 | 8.3 |
| 6 | Cyto-517 | 4.25 | 6.54 | 9.35 | 6.7 | 26 | 1843-461 | 5.5 | 8.35 | 11.1 | 8.3 |
| 7 | Cyto-313 | 4.51 | 5.98 | 9.45 | 6.6 | 27 | 1839-438 | 6.21 | 7.3 | 10.28 | 7.9 |
| 8 | Cyto-305 | 5.05 | 6.5 | 8.76 | 6.7 | 28 | 1840-441 | 7.13 | 7.89 | 10.2 | 8.4 |
| 9 | SL-516 | 8.8 | 9.15 | 12.79 | 10 | 29 | 1841-449 | 4.16 | 6.19 | 9.815 | 6.7 |
| 10 | Cyto-608 | 4.53 | 6.04 | 8.18 | 6.2 | 30 | 1859-558 | 4.63 | 5.15 | 8.115 | 5.9 |
| 11 | Cyto-164 | 4.68 | 6.79 | 8.4 | 6.6 | 31 | Tarzon-1 | 5.41 | 6.6 | 9.06 | 7.0 |
| 12 | FC-4245 | 4.95 | 6.23 | 9.4 | 6.8 | 32 | Stamp-81 | 4.54 | 6.25 | 9.4 | 6.7 |
| 13 | VH-305 | 4.25 | 6.78 | 9.45 | 6.8 | 33 | Stone ville-108 | 6 | 6.00 | 10.1 | 7.3 |
| 14 | CEMB-33 | 3.52 | 5.96 | 7.85 | 5.7 | 34 | Stone ville 15-17 | 3.55 | 5.23 | 7.36 | 5.3 |
| 15 | Chandni-95 | 4.95 | 6.6 | 9.73 | 7.0 | 35 | Ali Akber 802 | 4.83 | 5.9 | 9.84 | 6.8 |
| 16 | 1856-536 | 4.78 | 7.45 | 9.88 | 7.3 | 36 | Ali Akber 703 | 5.23 | 6.20 | 9.76 | 7.0 |
| 17 | 1852-511 | 3.55 | 5.0 | 6.67 | 5.0 | 37 | Delta pine-55 | 3.71 | 5.9 | 7.85 | 5.8 |
| 18 | 1855-533 | 6 | 6.5 | 10.0 | 7.5 | 38 | 4F | 5.56 | 6.75 | 9.55 | 7.2 |
| 19 | 1854-528 | 4.7 | 6.3 | 9.4 | 6.8 | 39 | CRIS-134 | 4.25 | 6.2 | 9.65 | 6.7 |
| 20 | 1845-472 | 5.41 | 6.6 | 9.01 | 7.01 | 40 | CRIS-508 | 4.98 | 6.8 | 8.79 | 6.86 |

**Table S 1.1 Mean values of Shoot length of cotton genotypes grown under three moisture levels.**

| **Sr.**  **No.** | **Genotypes**  **Name** | **Shoot length** | | | | **Sr.**  **No.** | **Genotypes**  **Name** | **Shoot length** | | | |
| --- | --- | --- | --- | --- | --- | --- | --- | --- | --- | --- | --- |
|  |  | **100%** | **75%** | **50%** | **Mean.**  **value** |  |  | **100%** | **75%** | **50%** | **Mean.**  **Value** |
| 1 | Cyto-178 | 12.18 | 7.57 | 4.58 | 8.11 | 21 | 1847-483 | 15.1 | 8.20 | 5.17 | 9.49 |
| 2 | Cyto-161 | 18.11 | 13.2 | 10.2 | 13.8 | 22 | 1863-577 | 16.86 | 9.65 | 7.93 | 11.4 |
| 3 | Cyto-179 | 15.08 | 10.70 | 8.65 | 11.4 | 23 | 1853-570 | 17.33 | 13.34 | 7.65 | 12.7 |
| 4 | Cyto-177 | 17.31 | 12.52 | 8.3 | 12.7 | 24 | 1866-598 | 14.23 | 8.685 | 6.61 | 9.84 |
| 5 | Cyto-124 | 19.52 | 12.73 | 7.48 | 13.2 | 25 | 1842-455 | 13.88 | 11.76 | 5.70 | 10.4 |
| 6 | Cyto-517 | 18.51 | 11.39 | 7.86 | 12.5 | 26 | 1843-461 | 17.53 | 11.76 | 7.74 | 12.3 |
| 7 | Cyto-313 | 16.36 | 10.16 | 6.58 | 11.0 | 27 | 1839-438 | 16.78 | 8.33 | 5.76 | 10.2 |
| 8 | Cyto-305 | 18.35 | 14.46 | 9.58 | 14.1 | 28 | 1840-441 | 13.73 | 14.3 | 7.45 | 11.8 |
| 9 | SL-516 | 18.63 | 14.5 | 10.3 | 14.4 | 29 | 1841-449 | 13.88 | 9.69 | 7.93 | 10.5 |
| 10 | Cyto-608 | 17.3 | 10.66 | 7.08 | 11.6 | 30 | 1859-558 | 16.86 | 8.74 | 5.06 | 10.2 |
| 11 | Cyto-164 | 15.99 | 9.85 | 6.91 | 10.9 | 31 | Tarzon-1 | 15.1 | 9.8 | 7.68 | 10.8 |
| 12 | FC-4245 | 16 | 11.29 | 7.46 | 11.5 | 32 | Stamp-81 | 15.91 | 10.33 | 7.25 | 11.1 |
| 13 | VH-305 | 15.35 | 9.93 | 6.6 | 10.6 | 33 | Stone ville-108 | 15.4 | 10.99 | 7.25 | 11.2 |
| 14 | CEMB-33 | 16.31 | 12.6 | 7.85 | 12.2 | 34 | Stone ville 15-17 | 17.93 | 7.73 | 4.18 | 9.95 |
| 15 | Chandni-95 | 18.28 | 9.76 | 7.26 | 11.7 | 35 | Ali Akber 802 | 12.19 | 8.88 | 6.59 | 9.22 |
| 16 | 1856-536 | 17.28 | 8.88 | 6.65 | 10.9 | 36 | Ali Akber 703 | 15.85 | 9.76 | 7.31 | 10.9 |
| 17 | 1852-511 | 18.14 | 8.06 | 4.08 | 10.0 | 37 | Delta pine-55 | 18.4 | 12.6 | 7.9 | 12.9 |
| 18 | 1855-533 | 15.9 | 11.1 | 7.2 | 11.4 | 38 | 4F | 15.3 | 10.1 | 6.5 | 10 |
| 19 | 1854-528 | 15.5 | 10.7 | 7.5 | 11.2 | 39 | CRIS-134 | 15.6 | 10.7 | 7.4 | 11 |
| 20 | 1845-472 | 15.9 | 9.8 | 7.7 | 11.1 | 40 | CRIS-508 | 16.2 | 9.35 | 7.0 | 10 |

**Table S 1.2 Mean values of RWC of cotton genotypes grown in three moisture levels.**

| **Sr. No.** | **Genotypes Name** | **RWC** | | | | **Sr. No.** | **Genotypes Name** | **RWC** | | | |
| --- | --- | --- | --- | --- | --- | --- | --- | --- | --- | --- | --- |
|  |  | **100%** | **75%** | **50%** | **Mean value** |  |  | **100%** | **75%** | **50%** | **Mean Value** |
| 1 | Cyto-178 | 66.3 | 49.0 | 33.16 | 49.5 | 21 | 1847-483 | 76.5 | 33.7 | 43.33 | 51.1 |
| 2 | Cyto-161 | 74 | 51.4 | 42 | 55.8 | 22 | 1863-577 | 79.8 | 63.5 | 44.66 | 62.6 |
| 3 | Cyto-179 | 74.8 | 55.1 | 48.17 | 59.3 | 23 | 1853-570 | 72 | 58.55 | 43.16 | 57.9 |
| 4 | Cyto-177 | 75.8 | 57.5 | 53.15 | 62.1 | 24 | 1866-598 | 74.8 | 58.5 | 44.16 | 59.1 |
| 5 | Cyto-124 | 75.3 | 58.1 | 47.16 | 60.1 | 25 | 1842-455 | 74.6 | 49.9 | 45 | 56.5 |
| 6 | Cyto-517 | 82 | 60.1 | 54.83 | 65.6 | 26 | 1843-461 | 85.5 | 49.0 | 36.5 | 57.0 |
| 7 | Cyto-313 | 75 | 60.6 | 47.31 | 60.9 | 27 | 1839-438 | 66.3 | 51.4 | 40.83 | 52.8 |
| 8 | Cyto-305 | 76.1 | 55 | 43 | 58.0 | 28 | 1840-441 | 74 | 57.1 | 44.83 | 58.6 |
| 9 | SL-516 | 80.5 | 59.5 | 56.83 | 65.6 | 29 | 1841-449 | 74.8 | 48.4 | 51 | 58.0 |
| 10 | Cyto-608 | 77.8 | 57.5 | 51.16 | 62.1 | 30 | 1859-558 | 75.8 | 58.1 | 48.5 | 60.8 |
| 11 | Cyto-164 | 75.1 | 59.5 | 40.83 | 58.4 | 31 | Tarzon-1 | 75.3 | 60.1 | 51 | 62.1 |
| 12 | FC-4245 | 76.6 | 60.0 | 42.83 | 59.8 | 32 | Stamp-81 | 82 | 60.6 | 50.67 | 64.4 |
| 13 | VH-305 | 74.3 | 51 | 51 | 58.7 | 33 | Stone ville-108 | 75 | 55 | 41.5 | 57.1 |
| 14 | CEMB-33 | 72.3 | 58.6 | 48.5 | 59.8 | 34 | Stone ville 15-17 | 76 | 59.5 | 43.16 | 59.5 |
| 15 | Chandni-95 | 75.1 | 59.5 | 51 | 61.8 | 35 | Ali Akber 802 | 80.5 | 57.5 | 42.5 | 60.1 |
| 16 | 1856-536 | 73.3 | 53.9 | 50.67 | 59.3 | 36 | Ali Akber 703 | 77.8 | 53.9 | 44 | 58.5 |
| 17 | 1852-511 | 71.3 | 40.7 | 41.5 | 51.1 | 37 | Delta pine-55 | 75.1 | 40.4 | 41.83 | 52.4 |
| 18 | 1855-533 | 74 | 61. | 44.1 | 59 | 38 | 4F | 76.6 | 61 | 44.6 | 60.7 |
| 19 | 1854-528 | 75 | 56 | 42.5 | 58 | 39 | CRIS-134 | 74.3 | 58.3 | 44.6 | 59 |
| 20 | 1845-472 | 73 | 53.6 | 49.56 | 56 | 40 | CRIS-508 | 73.4 | 57.2 | 45.5 | 58 |

**Table S 1.3 Mean values of ELWL of cotton genotypes grown under three moisture levels.**

| **Sr. No.** | **Genotypes Name** | **ELWL** | | | | **Sr. No.** | **Genotypes Name** | **ELWL** | | | |
| --- | --- | --- | --- | --- | --- | --- | --- | --- | --- | --- | --- |
|  |  | **100%** | **75%** | **50%** | **Mean. value** |  |  | **100%** | **75%** | **50%** | **Mean Value** |
| 1 | Cyto-178 | 0.56 | 0.36 | 0.42 | 0.4 | 21 | 1847-483 | 1.0 | 0.44 | 0.7 | 0.7 |
| 2 | Cyto-161 | 0.63 | 1 | 0.77 | 0.8 | 22 | 1863-577 | 1.9 | 0.79 | 1.0 | 1.2 |
| 3 | Cyto-179 | 1.05 | 0.53 | 0.56 | 0.7 | 23 | 1853-570 | 1.4 | 0.73 | 0.5 | 0.9 |
| 4 | Cyto-177 | 1.14 | 0.6 | 0.73 | 0.8 | 24 | 1866-598 | 0.7 | 0.61 | 0.4 | 0.6 |
| 5 | Cyto-124 | 1.32 | 0.47 | 0.91 | 0.9 | 25 | 1842-455 | 0.8 | 0.71 | 0.4 | 0.6 |
| 6 | Cyto-517 | 2.00 | 1.30 | 1.3 | 1.5 | 26 | 1843-461 | 0.7 | 0.55 | 0.5 | 0.6 |
| 7 | Cyto-313 | 0.83 | 0.71 | 0.6 | 0.7 | 27 | 1839-438 | 1.0 | 0.75 | 0.4 | 0.7 |
| 8 | Cyto-305 | 1.03 | 0.78 | 0.72 | 0.8 | 28 | 1840-441 | 0.9 | 0.65 | 0.5 | 0.7 |
| 9 | SL-516 | 1.91 | 1.39 | 1.1 | 1.4 | 29 | 1841-449 | 1.0 | 0.42 | 1.0 | 0.8 |
| 10 | Cyto-608 | 1.16 | 0.77 | 0.41 | 0.7 | 30 | 1859-558 | 1.4 | 1.27 | 0.3 | 1.0 |
| 11 | Cyto-164 | 0.86 | 0.75 | 0.68 | 0.7 | 31 | Tarzon-1 | 0.8 | 0.74 | 0.5 | 0.7 |
| 12 | FC-4245 | 0.71 | 0.75 | 0.69 | 0.7 | 32 | Stamp-81 | 0.8 | 0.61 | 0.5 | 0.6 |
| 13 | VH-305 | 0.87 | 0.78 | 0.64 | 0.7 | 33 | Stone ville-108 | 0.9 | 0.71 | 0.4 | 0.6 |
| 14 | CEMB-33 | 0.54 | 0.78 | 1.04 | 0.7 | 34 | Stone ville 15-17 | 0.8 | 0.49 | 0.4 | 0.5 |
| 15 | Chandni-95 | 1.91 | 1.20 | 1.1 | 1.4 | 35 | Ali Akber 802 | 0.4 | 0.26 | 0.3 | 0.3 |
| 16 | 1856-536 | 1.16 | 0.69 | 0.6 | 0.82 | 36 | Ali Akber 703 | 0.7 | 0.34 | 0.4 | 0.4 |
| 17 | 1852-511 | 0.67 | 0.595 | 0.36 | 0.54 | 37 | Delta pine-55 | 0.8 | 0.60 | 0.59 | 0.69 |
| 18 | 1855-533 | 0.915 | 0.555 | 0.515 | 0.66 | 38 | 4F | 0.89 | 0.65 | 0.52 | 0.68 |
| 19 | 1854-528 | 1.45 | 0.765 | 0.5 | 0.90 | 39 | CRIS-134 | 0.86 | 0.56 | 0.47 | 0.63 |
| 20 | 1845-472 | 0.765 | 0.65 | 0.56 | 0.65 | 40 | CRIS-508 | 0.98 | 0.51 | 0.44 | 0.64 |

**Table S 1.4 Mean values of POD of cotton genotypes grown under three moisture levels.**

| **Sr. No.** | **Genotypes Name** | **POD** | | | | **Sr. No.** | **Genotypes Name** | **POD** | | | |
| --- | --- | --- | --- | --- | --- | --- | --- | --- | --- | --- | --- |
|  |  | **100%** | **75%** | **50%** | **Mean value** |  |  | **100%** | **75%** | **50%** | **Mean value** |
| 1 | Cyto-178 | 13.5 | 14.5 | 10.5 | 12.83 | 21 | 1847-483 | 14.5 | 15 | 15.5 | 15 |
| 2 | Cyto-161 | 15 | 10 | 14.5 | 13.16 | 22 | 1863-577 | 12 | 14 | 10 | 12 |
| 3 | Cyto-179 | 12.5 | 14.5 | 15 | 14 | 23 | 1853-570 | 15.5 | 13 | 16 | 14.83 |
| 4 | Cyto-177 | 12.5 | 13.5 | 10 | 12 | 24 | 1866-598 | 11.5 | 15 | 22 | 16.16 |
| 5 | Cyto-124 | 14.5 | 15 | 14 | 14.5 | 25 | 1842-455 | 13 | 13 | 15.5 | 13.83 |
| 6 | Cyto-517 | 16.5 | 12.5 | 13.5 | 14.166 | 26 | 1843-461 | 16.5 | 17 | 13.5 | 15.66 |
| 7 | Cyto-313 | 13.5 | 15 | 16.5 | 15 | 27 | 1839-438 | 15 | 17.5 | 14.5 | 15.66 |
| 8 | Cyto-305 | 15 | 11 | 15 | 13.66 | 28 | 1840-441 | 12 | 15 | 13.5 | 13.5 |
| 9 | SL-516 | 11.5 | 13.5 | 15.5 | 13.5 | 29 | 1841-449 | 13.5 | 13 | 14.5 | 13.66 |
| 10 | Cyto-608 | 13.5 | 14 | 10 | 12.5 | 30 | 1859-558 | 12.5 | 16.5 | 11 | 13.33 |
| 11 | Cyto-164 | 16.5 | 16 | 13.5 | 15.33 | 31 | Tarzon-1 | 13 | 10.5 | 11.5 | 11.66 |
| 12 | FC-4245 | 13.5 | 13.5 | 12.5 | 13.16 | 32 | Stamp-81 | 15.5 | 14.5 | 16.5 | 15.5 |
| 13 | VH-305 | 10 | 12 | 10 | 10.66 | 33 | Stone ville-108 | 11.5 | 15 | 13.5 | 13.33 |
| 14 | CEMB-33 | 14.5 | 13 | 20 | 15.83 | 34 | Stone ville 15-17 | 12 | 13.5 | 15 | 13.5 |
| 15 | Chandni-95 | 16.5 | 14.5 | 14.5 | 15.16 | 35 | Ali Akber 802 | 16.5 | 11.5 | 13 | 13.66 |
| 16 | 1856-536 | 13.5 | 10.5 | 11.5 | 11.83 | 36 | Ali Akber 703 | 14.5 | 15.5 | 10.5 | 13.5 |
| 17 | 1852-511 | 14.5 | 11.5 | 13.5 | 13.16 | 37 | Delta pine-55 | 15 | 14.5 | 14.5 | 14.66 |
| 18 | 1855-533 | 10 | 12 | 16.5 | 12.83 | 38 | 4F | 12 | 13 | 13 | 12.66 |
| 19 | 1854-528 | 15 | 13.5 | 12.5 | 13.66 | 39 | CRIS-134 | 13.5 | 11.5 | 15 | 13.33 |
| 20 | 1845-472 | 12.5 | 15 | 14.5 | 14 | 40 | CRIS-508 | 16.5 | 16.5 | 14.5 | 15.83 |

**Table S 1.5 Mean values of H_2_O_2_ of cotton genotypes grown under three moisture levels.**

| **Sr. No.** | **Genotypes Name** | **H_2_O_2_** | | | | **Sr. No.** | **Genotypes Name** | **H_2_O_2_** | | | |
| --- | --- | --- | --- | --- | --- | --- | --- | --- | --- | --- | --- |
|  |  | **100%** | **75%** | **50%** | **Mean value** |  |  | **100%** | **75%** | **50%** | **Mean value** |
| 1 | Cyto-178 | 0.33 | 0.37 | 0.35 | 0.35 | 21 | 1847-483 | 0.145 | 0.15 | 0.24 | 0.18 |
| 2 | Cyto-161 | 0.205 | 0.33 | 0.31 | 0.28 | 22 | 1863-577 | 0.145 | 0.34 | 0.26 | 0.24 |
| 3 | Cyto-179 | 0.32 | 0.16 | 0.14 | 0.20 | 23 | 1853-570 | 0.14 | 0.12 | 0.23 | 0.16 |
| 4 | Cyto-177 | 0.325 | 0.14 | 0.12 | 0.19 | 24 | 1866-598 | 0.185 | 0.16 | 0.21 | 0.18 |
| 5 | Cyto-124 | 0.155 | 0.12 | 0.1 | 0.12 | 25 | 1842-455 | 0.355 | 0.18 | 0.23 | 0.25 |
| 6 | Cyto-517 | 0.165 | 0.36 | 0.36 | 0.29 | 26 | 1843-461 | 0.33 | 0.18 | 0.29 | 0.26 |
| 7 | Cyto-313 | 0.355 | 0.16 | 0.13 | 0.21 | 27 | 1839-438 | 0.185 | 0.14 | 0.25 | 0.19 |
| 8 | Cyto-305 | 0.135 | 0.25 | 0.20 | 0.19 | 28 | 1840-441 | 0.16 | 0.16 | 0.19 | 0.17 |
| 9 | SL-516 | 0.15 | 0.31 | 0.28 | 0.24 | 29 | 1841-449 | 0.15 | 0.36 | 0.29 | 0.26 |
| 10 | Cyto-608 | 0.33 | 0.18 | 0.16 | 0.22 | 30 | 1859-558 | 0.14 | 0.31 | 0.27 | 0.24 |
| 11 | Cyto-164 | 0.185 | 0.18 | 0.15 | 0.17 | 31 | Tarzon-1 | 0.165 | 0.19 | 0.23 | 0.19 |
| 12 | FC-4245 | 0.185 | 0.19 | 0.16 | 0.17 | 32 | Stamp-81 | 0.35 | 0.14 | 0.12 | 0.20 |
| 13 | VH-305 | 0.155 | 0.13 | 0.18 | 0.15 | 33 | Stone ville-108 | 0.175 | 0.13 | 0.15 | 0.15 |
| 14 | CEMB-33 | 0.145 | 0.14 | 0.19 | 0.16 | 34 | Stone ville 15-17 | 0.345 | 0.14 | 0.12 | 0.20 |
| 15 | Chandni-95 | 0.17 | 0.14 | 0.19 | 0.16 | 35 | Ali Akber 802 | 0.365 | 0.17 | 0.20 | 0.24 |
| 16 | 1856-536 | 0.125 | 0.34 | 0.29 | 0.25 | 36 | Ali Akber 703 | 0.15 | 0.18 | 0.17 | 0.16 |
| 17 | 1852-511 | 0.145 | 0.35 | 0.32 | 0.27 | 37 | Delta pine-55 | 0.34 | 0.37 | 0.23 | 0.31 |
| 18 | 1855-533 | 0.33 | 0.34 | 0.29 | 0.32 | 38 | 4F | 0.17 | 0.18 | 0.19 | 0.18 |
| 19 | 1854-528 | 0.35 | 0.38 | 0.41 | 0.38 | 39 | CRIS-134 | 0.135 | 0.14 | 0.19 | 0.15 |
| 20 | 1845-472 | 0.365 | 0.14 | 0.20 | 0.23 | 40 | CRIS-508 | 0.24 | 0.14 | 0.20 | 0.19 |

**Table S2. Factor loadings by various seedling traits in cotton genotypes at 100% moisture Level**

| **Variables** | **PC1** | **PC2** | **PC3** | **PC4** | **PC5** | **PC6** |
| --- | --- | --- | --- | --- | --- | --- |
| Eigen value | 1.8303 | 1.2816 | 1.1606 | 0.7560 | 0.5626 | 0.4089 |
| Total variance (%) | 0.305 | 0.214 | 0.193 | 0.126 | 0.094 | 0.068 |
| Cumulative variance (%) | 0.305 | 0.519 | 0.712 | 0.838 | 0.932 | 1.000 |
| Traits | | |  |  |  |  |
| RL | 0.395 | 0.573 | -0.112 | -0.429 | 0.248 | -0.508 |
| SL | 0.473 | -0.449 | 0.165 | 0.473 | 0.009 | -0.570 |
| RLWC | 0.359 | 0.393 | 0.531 | 0.104 | -0.614 | 0.218 |
| ELWL | 0.617 | -0.029 | -0.119 | 0.118 | 0.474 | 0.605 |
| POD | 0.064 | -0.454 | 0.580 | -0.655 | 0.150 | 0.037 |
| H_2_O_2_ | -0.328 | 0.330 | 0.573 | 0.372 | 0.561 | -0.049 |

**Table S3. Factor loadings by various seedling traits of cotton genotypes at 75% moisture Level**

| **Variables** | **PC1** | **PC2** | **PC3** | **PC4** | **PC5** | **PC6** |
| --- | --- | --- | --- | --- | --- | --- |
| Eigen value | 1.8793 | 1.1218 | 0.9421 | 0.8821 | 0.7224 | 0.4523 |
| Total variance (%) | 0.313 | 0.187 | 0.157 | 0.147 | 0.120 | 0.075 |
| Cumulative variance (%) | 0.313 | 0.500 | 0.657 | 0.604 | 0.925 | 1.000 |
| Trait | | |  |  |  |  |
| RL | 0.501 | -0.307 | 0.349 | -0.365 | 0.191 | 0.603 |
| SL | 0.529 | 0.078 | 0.527 | 0.129 | 0.058 | -0.645 |
| RLWC | 0.388 | 0.182 | 0.605 | 0.087 | 0.278 | 0.605 |
| ELWL | 0.463 | -0.031 | -0.223 | 0.442 | -0.701 | 0.219 |
| POD | -0.094 | -0.849 | -0.125 | -0.253 | -0.255 | -0.355 |
| H_2_O_2_ | -0.310 | -0.381 | 0.412 | 0.716 | 0.195 | 0.197 |

**Table S4. Factor loadings by various seedling traits of cotton genotypes grown at 50% moisture Level**

| **Variables** | **PC1** | **PC2** | **PC3** | **PC4** | **PC5** | **PC6** |
| --- | --- | --- | --- | --- | --- | --- |
| Eigen value | 1.9612 | 1.2170 | 1.0203 | 0.8696 | 0.5281 | 0.4038 |
| Total variance (%) | 0.327 | 0.203 | 0.170 | 0.145 | 0.088 | 0.067 |
| Cumulative variance (%) | 0.327 | 0.530 | 0.700 | 0.845 | 0.933 | 1.000 |
| Traits | | |  |  |  |  |
| RL | 0.263 | 0.317 | 0.785 | 0.001 | -0.460 | 0.040 |
| SL | 0.562 | 0.075 | 0.220 | -0.105 | 0.722 | -0.314 |
| RLWC | 0.490 | -0.071 | -0.343 | 0.521 | -0.393 | -0.459 |
| ELWL | 0.592 | -0.126 | -0.226 | -0.110 | -0.068 | 0.752 |
| POD | 0.107 | 0.611 | -0.392 | -0.613 | -0.207 | -0.208 |
| H_2_O_2_ | 0.115 | -0.707 | 0.112 | -0.573 | -0.256 | -0.282 |

**Table S5 Calculation of means for each cluster depending on traits variability at 100% moisture level**

| **Clusters Name** | **Traits** | | | | | | |
| --- | --- | --- | --- | --- | --- | --- | --- |
|  | **RL** | **SL** | **RWC** | **ELWL** | **POD** | **H2O2** |  |
| Cluster 1 | 8.8 | 18.6 | 80.5 | 1.91 | 11.5 | 0.15 |  |
| Cluster 2 | 4.79 | 16 | 82.5 | 1.01 | 16.25 | 0.3 |  |
| Cluster 3 | 5.12 | 15.6 | 74.1 | 0.89 | 10 | 0.24 |  |
| Cluster 4 | 5.62 | 16.2 | 77.9 | 1.08 | 12.9 | 0.23 |  |
| Cluster 5 | 4.45 | 16.5 | 74.2 | 0.98 | 13.5 | 0.18 |  |
| Cluster 6 | 4.84 | 15.9 | 75.1 | 1.06 | 13.76 | 0.22 |  |
| Cluster 7 | 5.08 | 17.1 | 73.9 | 0.91 | 14.5 | 0.18 |  |
| Cluster 8 | 5.3 | 14.4 | 66.3 | 0.81 | 14.25 | 0.25 |  |

Table S6 Calculation of means for each cluster depending on traits variability at 75% moisture level

| **Clusters** | **RL** | **SL** | **RWC** | **ELWL** | **POD** | **H2O2** |
| --- | --- | --- | --- | --- | --- | --- |
| Cluster 1 | 5.2 | 8.2 | 33.7 | 0.44 | 15 | 0.15 |
| Cluster 2 | 5.6 | 9.4 | 34.1 | 0.51 | 13 | 0.36 |
| Cluster 3 | 8.03 | 13.8 | 35.4 | 0.51 | 10.9 | 0.32 |
| Cluster 4 | 6.04 | 9.2 | 60.2 | 0.49 | 13 | 0.22 |
| Cluster 5 | 5.9 | 8.4 | 36.2 | 0.60 | 11.2 | 0.20 |
| Cluster 6 | 6.1 | 8.4 | 38.9 | 0.59 | 13.7 | 0.28 |
| Cluster 7 | 6.2 | 13.8 | 34.5 | 0.89 | 10.5 | 0.21 |
| Cluster 8 | 7.0 | 11.0 | 35.1 | 0.63 | 11.1 | 0.17 |

**Table S7. Calculation of means for each cluster depending on traits variability at 50 % moisture level**

| **Clusters** | **Traits** | | | | | |
| --- | --- | --- | --- | --- | --- | --- |
|  | **RL** | **SL** | **RWC** | **ELWL** | **POD** | **H2O2** |
| Cluster 1 | 12.79 | 14.4 | 56.8 | 1.1 | 15.5 | 0.28 |
| Cluster 2 | 9.19 | 7.8 | 51.9 | 0.7 | 11.08 | 0.22 |
| Cluster 3 | 9.5 | 7.4 | 49.1 | 0.8 | 15.1 | 0.16 |
| Cluster 4 | 7.9 | 7.2 | 46.3 | 0.7 | 21 | 0.20 |
| Cluster 5 | 9.1 | 6.7 | 45.7 | 0.6 | 10.5 | 0.23 |
| Cluster 6 | 9 | 6.1 | 42.7 | 0.5 | 13.7 | 0.21 |
| Cluster 7 | 9.3 | 7.6 | 43.6 | 0.5 | 14.7 | 0.22 |
| Cluster 8 | 8.4 | 5.9 | 34.8 | 0.4 | 12 | 0.32 |
